# Exploring the Determinants of Polypharmacy Prescribing and Dispensing Behaviours in Primary Care for the Elderly—Protocol for a Qualitative Study

**DOI:** 10.3390/ijerph18147656

**Published:** 2021-07-19

**Authors:** Najwa Taghy, Linda Cambon, Caroline Boulliat, Olivier Aromatario, Claude Dussart

**Affiliations:** 1 Laboratory P2S (Systemic Health Process), University Claude Bernard of Lyon 1, University of Lyon, EA4129 Lyon, France; 2 ISPED, U1219 Inserm Center, Bordeaux Population Health, University of Bordeaux, CHU Bordeaux, 33000 Bordeaux, France; linda.cambon@u-bordeaux.fr (L.C.); olivier.aromatario@u-bordeaux.fr (O.A.); 3 HIA Desgenettes, Pharmacy, 69000 Lyon, France; caroline.boulliat@intradef.gouv.fr; 4 Lyon Public Hospices, Central Pharmacy, Laboratory P2S (Systemic Health Process), University Claude Bernard Lyon 1, University of Lyon, EA4129 Lyon, France; claude.dussart@univ-lyon1.fr

**Keywords:** polypharmacy, aging, qualitative study, intervention, prescribing, dispensing, theoretical domains framework

## Abstract

Polypharmacy is becoming increasingly common, especially among the elderly. It often has a negative connotation, but is sometimes necessary or even desirable, and needed to categorize polypharmacy as appropriate or inappropriate. The challenge is in ensuring that this is considered appropriate when necessary. We aimed to develop an evidence-based intervention to reduce the risks associated with using a systematic approach, involving key stakeholders in prescribing and dispensing drugs to the elderly in primary care. The purpose of this study is to identify the key components which are perceived as influencing these behaviours. It is a qualitative study of general practitioners (GPS) and community pharmacists involved in the care of the elderly. The main inclusion criterion is the geographic location. Qualitative data will be generated from one-on-one, semi-structured interviews and processed for thematic content analysis. Our approach integrates the patient pathway in primary care. It considers the fact that GP and pharmacist behaviours are far from being independent. This study represents the first step in the process of developing an intervention theory which involves a crossover between data from the literature and the knowledge of experts, allowing us to interrogate hypotheses about the influences and mechanisms associated with prescribing and dispensing drugs to the elderly in primary care.

## 1. Introduction

The use of medications is increasing, especially among the elderly. This increase is mainly attributed to the high prevalence of multi-morbidity, with 50% of adults aged 75 and older now living with at least three concomitant conditions [1] and the broadening of therapeutic options, with an increasing number of clinical practice recommendations calling for the use of more than one drug in the management of chronic diseases [2,3,4]. Polypharmacy has been identified as the main determinant of potentially inappropriate prescribing among the elderly. It is associated with many negative clinical consequences caused by adverse drug events, but also with the decreased efficacy of treatments or a decreased adherence to treatments. Polypharmacy also has economic implications, including the cost of reimbursing potentially ineffective treatments and the cost of cascading complications [5,6].

There is no universally accepted definition of the term “polypharmacy”. Much of the literature to date has referred to numerical thresholds, such as the concomitant prescription of four or five drugs. However, recent studies have highlighted the need to take the clinical context in which drugs are prescribed into account. Therefore, the concept of inappropriate use of multiple medications should be preferred over the existing threshold-based definitions [7]. Appropriate polypharmacy is defined as “prescribing for individuals with complex or multiple conditions where drug use has been optimized and prescribing is consistent with the best available evidence”. The concept of “inappropriate multiple medications”, therefore, recognizes that patients can benefit from multiple medications if the prescription is evidence-based and reflects the clinical needs of the patient [5]. However, these definitions are still based on relatively imprecise concepts, such as “evidence-based” or “clinical needs”. A recent review identifies studies the literature that have defined appropriate or rational polypharmacy, or have recognized the distinction between appropriate and inappropriate medications [8]. These studies either defined polypharmacy using a brief description only, or used a brief description and polypharmacy tools, such as the Beers criteria and the Medication Appropriateness Index (MAI) [9,10,11,12,13,14]. Nevertheless, none of these definitions explicitly allow us to identify any list of behaviours classified as ‘inappropriate’ in terms of prescribing and dispensing drugs. Therefore, to achieve appropriate medication for the elderly, our approach must be multidimensional. This requires the merging of different disciplines because inappropriate polypharmacy is the result of the combination of several factors, which go far beyond the drug’s appropriateness [15].

Our hypothesis is that, in these complex situations, an intervention targeting the main determinants of inappropriate polypharmacy could reduce the associated risks. As such, we chose to investigate the types of approach that could improve the process of prescribing and dispensing medication for the elderly when their state requires more than one medication to be taken. We are specifically interested in the behaviour of general practitioners (GPs) and pharmacists, who, as primary health care providers, are poised to play a pivotal role mitigating this growing issue.

In France, both GPs and community pharmacists are considered as primary care providers. Since 2005, GPs have been given a soft gatekeeper role. Patients must designate a médecin traitant, who is a GP in the vast majority of cases [16,17,18]. Said GP will be their first point of contact with the health care system. He/she provides referrals to other health professionals (i.e., specialist doctors, providing ambulatory and hospital-based care) and seeks to develop a person-centered approach, ensuring continuous and long-term care [19]. Community pharmacists dispense medication and provide advice. As of March of 2018, community pharmacists have been mandated to support elderly people who are prescribed multiple medications, in close collaboration with GPs. The implementation of “Bilan partagé de médication”, which consists of a structured critical analysis of the patient’s medications with the objective of establishing a consensus with the patient regarding his or her treatment, taking care to optimize the clinical impact of the medications, reduce the number of problems related to the therapy, and reduce unnecessary extra costs, has truly formalized the shared follow-up of the elderly [20,21].

The research protocol described within is part of a broader research project with the aim of designing and developing an evidence-based, and inherently complex, intervention to reduce the risks associated with inappropriate polypharmacy. Our research project is organized in several phases. The purpose of this one is to develop a theory-based intervention using a systematic approach guided by the best available evidence and appropriate theory, and involving key stakeholders involved in prescribing and dispensing drugs to elderly patients in primary care (i.e., GPs and community pharmacists). The aim of this specific study is to identify and select the key components (mechanisms and activities) perceived as influencing the prescription and dispensing of appropriate polypharmacy.

## 2. Materials and Methods

### 2.1. The Underlying Theoretical Model

Public health, as well as other disciplines, has grasped the concept of complexity. This consideration, more instrumental than conceptual, of this notion in the field of health extends to the design and evaluation of so-called complex interventions. They are complex because they are supposed to act on behaviours that cannot be dissociated from their contexts or the systems to which they belong [22]. The Medical Research Council (MRC) emphasizes, according to the same principle, that an intervention is made up of several components that interact with each other and the context in which they take place. The MRC proposes the use of theory-based approaches for the evaluation and development of interventions [23,24,25]. 

Our approach has been modelled on these recommendations, and the underlying theoretical model is the theoretical domains framework (TDF). The TDF is founded in the psychological theory of behaviour change. By simplifying these concepts, researchers from non-health-psychology backgrounds can access behavioural theories [26]. Two versions exist: one with 12 theoretical domains and a second, more recent, and refined version with 14 domains [27]. In the appendix (i.e., Appendix A), a flow chart illustrates the two different versions of TDF. The first version of the TDF remains the most widely used version. This is the one we have chosen for our study [28,29,30,31]. Thus, the 12 theoretical domains that make up the TDF (v 1.0) and are relevant to changing behavioural health care professionals are: knowledge; skills; social/professional role and identity; beliefs about capabilities; beliefs about consequences; motivation and goals; memory, attention and decision processes; environmental context and resources; social influences; emotion; behavioural regulation; nature of the behaviours [26]. For our intervention on targeting prescribing and dispensing behaviour, the TDF v 1.0 will be used to identify key theoretical domains (mechanisms of interactions) that are perceived to influence providers’ behaviours and provide a solid evidence base to guide the intervention design. The key components will then be correlated with the behaviour change techniques (BCTs) that may form the “active ingredients” of the intervention (activities) using the S. Michie taxonomy [32].

### 2.2. Study Design

This is a qualitative study. Data will be collected through individual semi-directive interviews. This method of data collection is used to collect information from individuals about their own practices, beliefs, or opinions, to gather information on past or present behaviours or experiences and to tap into the expert knowledge of an individual. These interviews will likely gather factual material and data, such as descriptions of processes [33].

Individual semi-structured interviews also allow professionals the freedom to express their views on their own terms. It is also an appropriate data collection method when seeking to explore the perception and representation of GPs and pharmacists regarding polypharmacy use in the elderly, where our analysis will aim to explore their approaches to prescribing and dispensing polypharmacy in this age group, and their perceptions of barriers and facilitators to achieving appropriate polypharmacy in elderly patients in ambulatory care.

Semi-directive interview guides based on the 12 domains of the TDF will be used. Open-ended questions will be favored, in order to obtain individualized answers while ensuring that the themes covering the 12 domains are well addressed. The interviews will be conducted by the doctoral student (NT). Interviews will be recorded and then transcribed into a text file. An analysis of qualitative data will be carried out in collaboration with other members of the research team. 

Data collection for this study commenced in November 2020, and analyses are planned to be completed by the end of July 2021.

### 2.3. Constitution of the Panel

With the objective of understanding reality, our goal is not to establish a representative sample, but rather to identify individuals who possess relevant characteristics that allow us to explore the aspects of the studied phenomenon in depth [34].

The representation that interests us in this qualitative study is a representation of the perspectives, meanings, opinions and ideas of general practitioners and pharmacists with regard to the subject under study. The selected panel does not concern a population composed of individuals, but a phenomenon, the lived meaning of which we want to identify [35].

#### 2.3.1. Selection Criteria

To serve the objective of our qualitative study, the essential characteristics are, therefore, intentionality and pertinence to our research question [34]. Based on the idea of approaching “competent” GPs and pharmacists, who are likely to provide the most relevant information with regard to our initial questions and to capture the variability in the discourse on the subject under investigation, which is prescribing and dispensing drugs to the elderly in primary care, the main selection criterion was the geographic location of the medical office and pharmacies in which the GPs and pharmacists practice. We aimed to target different geographical areas with a large population of elderly people (aged 60 and over).

#### 2.3.2. Target Population

Data from The French National Institute of Statistics and Economic Studies (INSEE) describe the populations of different regions according to age. Nouvelle Aquitaine is the oldest region in France, with 29% of people aged 60 or over (compared with the national average of 25%); almost all of these seniors (95%) live at home [36]. Based on the available INSEE data, seven areas (i.e., Table 1) with an aging population, specifically municipalities where the proportion of the population aged 60 or over exceeds 40%, were targeted [37].

The constitution of the panel of providers will be conducted in two steps.

#### 2.3.3. Recruitment of General Practitioners

GPs were identified in the geographical areas previously defined using the Yellow Pages. Lists will be drawn up and a random selection of doctors to contact will be made. An initial contact will be established by phone. NT will provide potential participants with a brief overview of the study, and answer any questions. Thereafter, an invitation letter and an information sheet with a summary of the project will be sent to those who expressed a wish to receive additional information. Finally, the interviewer (NT) will find the most convenient place and time for a face-to-face interview with the GPs who consented. If necessary, particularly in the context of the COVID-19 epidemic, the interviews may also be conducted over the phone.

#### 2.3.4. Recruitment of Dispensing Pharmacists

The choice was made to select pharmacists in a second stage from the network of recruited GPs. In this manner, the recruited physicians will be asked to identify the local pharmacies that dispense most of the prescriptions for elderly patients in the practice. In the same way as for GPs, pharmacists will be contacted by phone by NT, who will provide a brief summary of the project and send additional information to those who have requested it. Appointments will be made, and interviews will be conducted, either in person or over the phone, with consenting pharmacists.

In all cases, written, informed consent will be obtained from all subjects.

#### 2.3.5. Panel Size

An initial panel size is estimated, but the size of the final panel will be determined by data saturation, meaning that no new information or themes emerge from the data and that the interview n + 1 does not provide anything new compared to the interview n [38,39,40]. This is implied in the protocol to transcribe and code the interviews as they are conducted. In total, there will be two groups, containing 14–20 GPs (ideally two and at least one from each of the seven zones) and 7–10 pharmacists (at least one from each of the seven zones). This will provide a total panel of 21–30 participants as an initial estimation. However, practical issues such as time or resource constraints, ethical precautions, or accessibility of the professionals to be interviewed may influence the final configuration of our panel.

### 2.4. Data Collection

#### 2.4.1. Topic Guides

A first phase of the research project, preceding this survey, consisted of exploring the existing scientific evidence, namely from other countries, and contextual and experiential knowledge about proven or promising interventions related to the issue of inappropriate polypharmacy.

More specifically, a large multi-phase research project has been conducted since 2015 by an Irish team, focusing on the development of interventions using a combination of evidence and theory to address inappropriate polypharmacy in the elderly [28]. One phase of this project resulted in the development of two interview guides based on the 12 domains of TDF, one for GPs (to study prescribing behaviour) and anther one for pharmacists (to study dispensing behaviour). Each topic guide includes a series of similar questions covering four key areas: professionals’ views on the term ‘polypharmacy’; professionals’ assessment of a clinical scenario depicting an older patient receiving inappropriate polypharmacy; professionals’ perceptions of barriers and facilitators to ensuring the prescribing (GPs) and dispensing (community pharmacists) of appropriate polypharmacy to older people; professionals’ views on potential intervention components and outcome measures for inclusion in future intervention studies. These interview guides were tested, validated, and used during the survey conducted by the Irish research team and published with the results of the survey [41].

Our strategy was to use these guides validated by a researchers’ consensus and to adapt them to the needs of the present survey in France. The adaptation of these guides was not limited to translation into French. It also consisted of modifying the questions to be compatible with the context of France. A first version of these two adapted interview guides was elaborated by NT and discussed with the fellow researchers. They were piloted beforehand, during one-on-one interviews with two doctors and two pharmacists who are not part of the sample. If required, questions were reworded, clarified, or completed in order to obtain final versions.

#### 2.4.2. Interview Process

NT will conduct semi-structured interviews with recruited professionals either at their place of work, at another convenient location or by phone. Interviews take about 45 min.

A presentation is scheduled at the beginning of each interview and the agreement of the participant will be obtained before starting.

Interviews will be audio-recorded in full using a voice recorder, and additional information will be noted manually. At the end of the interview, an open-ended question will be asked at in order to collect any comments from the participants about the interview process. At the end of the interview, and as soon as possible, non-verbal aspects will be noted, as well as general impressions of the interview process. A verbatim transcript will be made to faithfully reflect the entire content of the interview. The quality of the transcription will be checked for each recording. Recordings will be separated into two groups to distinguish the interviews of the doctors from those of the pharmacists, before being anonymized.

### 2.5. Analysis

We will employ thematic content analysis. The process of content analysis allows for the replacement of an intuitive or instinctive interpretation of a given message with a constructed one [42]. More specifically, a thematic analysis will be more conducive to our research because it is about identifying, in the multiform and varied content of an interview, the core of the meanings, to obtain a unique and original analysis of the themes we will have defined beforehand, and possibly specified by professional discourse [43].

NVivo software will be used to facilitate data analysis. There will be a stage where the transcripts are read and reread, without listening to the interview recordings. The transcripts will first be read, checked, and analysed by the NT and another member of the research team, as a validity check, will independently analyse at least 25% of the transcripts.

A two-step analysis will be adopted. First, a deductive approach using pre-defined coding categories. Second, an inductive approach in which the emerging content themes relating to barriers and facilitators within each domain were identified. The output from the analysis should allow us to identify key theoretical domains and select BCTs.

### 2.6. Data Protection and Ethics

Audio-recordings will be anonymized, with all identifiable information removed prior to using the software analysis tool. All audio-recordings will be destroyed immediately after transcription and the data will be stored on a server at the University of Lyon 1 according to the current standards and conditions. Ethical approval for this study protocol was obtained from the Ethics Committee of the University College of General Medicine (CUMG, University of Lyon, n°IRB: 2 July 2020).

## 3. Discussion

Close collaboration between general practitioners and pharmacists in primary care is one of the cornerstones of the multidisciplinary approach, which is recommended in order to optimize the care of the elderly. In France, cooperation between healthcare professionals is a major incentive of the current French national health strategy, “Ma santé 2022”, launched in September 2018. Improved collaboration between healthcare providers aims to optimize care pathways, and thus meet the expectations of both patients and providers [44,45]. With regards to the collaboration between GPs and pharmacists, various programs, initiated by the providers or of governmental origin, such as the PAERPA program (Parcours des personnes âgées en risque de perte d’autonomie—care pathways for older people at risk of loss of autonomy)*,* have led to the development of collaborative processes which bring GPs into contact with pharmacists in the context of daily inter-professional communication [46,47]. This collaboration is promoted and reinforced by tools such as the medication review (Bilan partagé de médication) which, even more specifically, involves a collaboration between GPs and pharmacists for the management of elderly people experiencing polypharmacy [21]. Although this collaboration remains, in fact, largely informal, we have intended to capitalize on it in the design of this formative research study by deliberately recruiting pharmacists from the network of previously recruited physicians. Although our analysis approach will not take strict physician/pharmacist pairs into account, this recruitment strategy allows for a selection of providers involved in the care, to a certain extent, of the same elderly population. As the two behaviours, prescribing and dispensing drugs, are far from being independent of each other, we have considered the patient pathway in our approach.

## 4. Conclusions

This step of identifying and selecting the key mechanisms perceived to influence the prescribing and dispensing of appropriate polypharmacy is a building block in the process of developing an intervention theory targeting these behaviours. This process involves crossing data from the literature and the knowledge of experts (GPs and pharmacists in our case) in a structured approach. The review of the literature can find evidence and define hypotheses. The objective of crossing this with the expertise of professionals is to verify these hypotheses, explaining which components (activities) can influence the targeted behaviours and which mechanisms they use, and to adapt these hypotheses according to the context. The finalized intervention theory (the expected outcome) will consist of an analytical grid composed of x activities (BCTs), which activate x mechanisms influencing the behaviour of GPs (prescription of appropriate polypharmacy for the elderly) and pharmacists (dispensing of appropriate polypharmacy for the elderly).

## Figures and Tables

**Table 1:** Selected Areas in Nouvelle-Aquitaine.

Areas	Municipalities	Departments
Area 1	Arcachon	Gironde
Area 2	Soulac-sur-Mer, Le Verdon, Arès	Gironde
Area 3	Biarritz	Pyrénées-Atlantiques
Area 4	Soorts-Hossegor	Landes
Area 5	Cantons d’Eymet, St-Alvère et Domme	Dordogne
Area 6	Cantons de Lauzun, Villeréal, Castillonnès	Lot-et-Garonne
Area 7	Cantons de Hautefort, Jumilhac-Le-Grand, St-Pardoux-La-Rivière, Bussière-Badil, Mareuil, Verteillac, Saint-Aulaye	Dordogne

## Data Availability

This article does not data and the data availability policy is not applicable.
